# Sex-Disaggregated Data on Clinical Characteristics and Outcomes of Hospitalized Patients With COVID-19: A Retrospective Study

**DOI:** 10.3389/fcimb.2021.680422

**Published:** 2021-05-26

**Authors:** Mengdie Wang, Nan Jiang, Changjun Li, Jing Wang, Heping Yang, Li Liu, Xiangping Tan, Zhenyuan Chen, Yanhong Gong, Xiaoxv Yin, Qiao Zong, Nian Xiong, Guopeng Zhang

**Affiliations:** ^1^ Department of Neurology, Union Hospital, Tongji Medical College, Huazhong University of Science and Technology, Wuhan, China; ^2^ Department of Social Medicine and Health Management, School of Public Health, Tongji Medical College, Huazhong University of Science and Technology, Wuhan, China; ^3^ Department of Neurology, The Central Hospital of Wuhan, Tongji Medical College, Huazhong University of Science and Technology, Wuhan, China; ^4^ School of Nursing, Wuchang University of Technology, Wuhan, China; ^5^ Office of Academic Research, The Central Hospital of Wuhan, Tongji Medical College, Huazhong University of Science and Technology, Wuhan, China; ^6^ Lichuan Center for Disease Control and Prevention, Lichuan, China; ^7^ Department of Nuclear medicine, Tongji Hospital, Tongji Medical College, Huazhong University of Science and Technology, Wuhan, China

**Keywords:** COVID-19, SARS-CoV-2, sex, menopause, estrogen, China

## Abstract

**Background:**

Sex and gender are crucial variables in coronavirus disease 2019 (COVID-19). We sought to provide information on differences in clinical characteristics and outcomes between male and female patients and to explore the effect of estrogen in disease outcomes in patients with COVID-19.

**Method:**

In this retrospective, multi-center study, we included all confirmed cases of COVID-19 admitted to four hospitals in Hubei province, China from Dec 31, 2019 to Mar 31, 2020. Cases were confirmed by real-time RT-PCR and were analyzed for demographic, clinical, laboratory and radiographic parameters. Random-effect logistic regression analysis was used to assess the association between sex and disease outcomes.

**Results:**

A total of 2501 hospitalized patients with COVID-19 were included in the present study. The clinical manifestations of male and female patients with COVID-19 were similar, while male patients have more comorbidities than female patients. In terms of laboratory findings, compared with female patients, male patients were more likely to have lymphopenia, thrombocytopenia, inflammatory response, hypoproteinemia, and extrapulmonary organ damage. Random-effect logistic regression analysis indicated that male patients were more likely to progress into severe type, and prone to ARDS, secondary bacterial infection, and death than females. However, there was no significant difference in disease outcomes between postmenopausal and premenopausal females after propensity score matching (PSM) by age.

**Conclusions:**

Male patients, especially those age-matched with postmenopausal females, are more likely to have poor outcomes. Sex-specific differences in clinical characteristics and outcomes do exist in patients with COVID-19, but estrogen may not be the primary cause. Further studies are needed to explore the causes of the differences in disease outcomes between the sexes.

## Introduction

Coronavirus disease 2019 (COVID-19) has been reported in 223 countries and regions, with a cumulative total of 131,837,512 confirmed cases and 2,862,664 deaths as of April 7, 2021, according to the World Health Organization. COVID-19 pandemic has posed a serious threat to global health and economy. Severe acute respiratory syndrome coronavirus 2 (SARS-CoV-2) is the pathogen that causes COVID-19 *via* using angiotensin converting enzyme 2 (ACE2) as a receptor (Zhao et al., 2020). It is a member of the beta coronavirus genus along with severe acute respiratory syndrome coronavirus (SARS-CoV) and Middle East respiratory syndrome coronavirus (MERS-CoV) (DiMaio et al., 2020). The typical clinical manifestations of patients with COVID-19 are fever, dry cough, fatigue, and in severe cases, dyspnea (Guan et al., 2020; Hu et al., 2021). In previous epidemics of coronavirus-induced diseases, infected populations have shown gender differences in clinical outcomes. Studies related to SARS-CoV showed that males were more susceptible to infection and had a significantly higher case fatality rate than females (Karlberg et al., 2004; Leung et al., 2004; Leong et al., 2006). Among patients infected by MERS-CoV, morbidity and mortality were higher in males than females (Alghamdi et al., 2014). Similar to these respiratory diseases caused by coronaviruses, sex and gender are crucial variables in COVID-19 (Channappanavar et al., 2017; Kadel and Kovats, 2018; Guan et al., 2020). Epidemiologic data suggested that males may be more susceptible to COVID-19 and have higher clinical severity and mortality, especially older males with chronic illnesses (Epidemiology Working Group for NCIP Epidemic Response and Chinese Center for Disease Control and Prevention, 2020; Guan et al., 2020; Onder et al., 2020). Such sex-specific differences are probably attributed to sex hormones, different copy numbers of immune response genes on X chromosomes, and the presence of disease susceptibility genes in females and males (Sue, 2017; Kadel and Kovats, 2018; Schurz et al., 2019). Behavioral and cultural factors may also be involved (Suen et al., 2019; Cai, 2020).

Sex- and gender- specific epidemiologic observations would help deepen our understanding towards COVID-19 and make sex- or gender- specific recommendations (Bhopal, 2020; Wenham et al., 2020). Although current studies have reported the impact of sex on patients with COVID-19, studies based on Chinese populations are scarce and limited in small sample sizes (Sha et al., 2021). Estrogen has been reported to play a crucial role in disease outcomes in COVID-19 patients, attributed to its ability to reduce inflammatory IL-6, IL-8 and TNF-α levels (Alwani et al., 2021). However, this conclusion is not consistent (Alwani et al., 2021; Sha et al., 2021). This study aimed to provide information on differences in clinical characteristics and outcomes between male and female patients and explore the effect of estrogen on disease outcomes.

## Materials and Methods

### Study Design and Participants

This multi-center retrospective study analyzed information on hospitalized patients with COVID-19 admitted to four hospitals (the Central Hospital of Wuhan, Wuhan Red Cross Hospital, the Central Hospital of Enshi Tujia and Miao Autonomous Prefecture and Lichuan People’s Hospital) in Hubei Province, China. All these hospitals are government-appointed hospitals dedicated to the treatment of COVID-19. The diagnosis of COVID-19 was confirmed according to the WHO interim guidance and the Diagnosis and Treatment Protocol for Coronavirus Pneumonia (trial version 7) released by National Health Commission of China (National Health Commission of the People’s Republic of China; World Health Organization). A total of 2501 patients with COVID-19 admitted to these hospitals from 31st December 2019 to 31st March 2020 were enrolled. This study was approved by the Medical Ethics Committee of Tongji Medical College, Huazhong University of Science and Technology (Number: 2020IECA252) and complied with the principles of the Declaration of Helsinki. The requirement for informed consent from patients was waived by the ethics committee due to the retrospective nature of the study. The data are anonymous, and all authors could only use the anonymized data for statistical analysis, with no direct interaction with patients or patient samples.

### Data Collection

The demographic characteristics, medical history, laboratory findings, chest computed tomography (CT) on admission, and outcome data were extracted from electronic medical records. Laboratory assessments comprised complete blood count, coagulation test, biochemical test (including liver and renal function, cardiac enzymes), and infection-related indices. The laboratory findings presented in the study were collected at hospital admission of COVID-19 patients. The primary outcome variables of the study were disease severity and in-hospital mortality. The secondary outcomes were complications, which included shock, acute respiratory distress syndrome (ARDS), acute cardiac injury, acute kidney injury (AKI), secondary bacterial infection, and urinary tract infection. ARDS and shock were defined in term of the interim guidance of WHO for novel coronavirus (World Health Organization). Cardiac injury was diagnosed when serum levels of cardiac biomarkers (e.g., high-sensitivity troponin I, hs-TNI) were above the 99th percentile upper reference limit (Shi et al., 2020). Acute kidney injury was identified according to the kidney disease: Improving Global Outcomes definition (Kellum et al., 2012). Secondary bacterial infection was confirmed if the patients had symptoms or signs of nosocomial pneumonia or bacteremia, and a positive culture of a new pathogen from a lower respiratory tract specimen or from blood samples taken after admission (Garner et al., 1988). Urinary tract infection was determined by an abnormally elevated leukocyte count in the urine (Long and Koyfman, 2018). The severity of COVID-19 (severe *vs.* non-severe) was assessed at admission according to the Diagnosis and Treatment Protocol for Coronavirus Pneumonia (trial version 7) released by National Health Commission of China (National Health Commission of the People’s Republic of China). The clinical outcomes were categorized into discharges and mortality and monitored up to Apr 5, 2020.

To explore the effects of estrogen, female patients with COVID-19 were divided into two groups (premenopausal and postmenopausal female patients) according to whether they are menopausal, and male ones were classed into two groups based on their age (male patients <50 years and ≥50 years), which refer to the median age of female menopause.

### Statistical Analysis

In this study, continuous variables were represented by median and interquartile range (IQR), and categorical variables were presented as frequencies and percentage (%). Comparison of parameters between two groups were conducted with the Wilcoxon-Mann-Whitney-Test for continuous variables. Pearson’s χ^2^ test or Fisher’s exact tests were used for categorical variables. The risk of outcomes of interest was calculated by multivariable logistic regression. Hospital was modeled as a random effect in the random effect logistic regression. Adjusted odd ratios (ORs) and 95% confidence intervals (CIs) were calculated for different groups. Multivariate analyses were all adjusted for age and comorbidities (hypertension, diabetes, coronary heart disease, cerebrovascular disease, COPD, malignancy, chronic liver disease, and chronic kidney disease). Propensity score-matched analysis was used to balance the age between premenopausal and postmenopausal females. Two cohorts were matched at a ratio of 1:1 with a caliper width of 0.2. For all comparisons, differences were tested using two-tailed tests and p-values less than 0.05 were considered statistically significant. Statistical analysis was performed using SAS version 9.4.

## Results

### Clinical Characteristics and Radiographic Parameters of Female and Male Patients

A total of 2501 hospitalized patients with COVID-19 were included in the analysis. Of these, 1305 were females and 1196 were males. The features of the study population are shown in **Table 1**. The median age was 56 years (IQR, 40-67 years) for females and 59 years (IQR, 44-69 years) for males. The clinical manifestations of male and female patients were similar. Fever and dry cough were the major common symptoms, whereas myalgia, diarrhea, and vomiting were rare. Fever was present more in male patients (795[66.5%] *vs.* 806[61.8%], *P*=0.0142). Diarrhea was present more in female patients (75[5.7%] *vs.* 45[3.8%], *P*=0.0204). Male patients had more comorbidities, including hypertension (457[38.2%] *vs.* 418[32.0%], *P*=0.0012), coronary heart disease (145[12.1%] *vs.* 90[6.9%], *P*<0.0001), cerebrovascular disease (107[8.9%] *vs.* 62[4.8%], *P*<0.0001), COPD (109[9.1%] *vs.* 64[4.9%], *P*<0.0001), and chronic kidney disease (87[7.3%] *vs.* 50[3.8%], *P*=0.0002).

**Table 1:** Clinical characteristics on admission and outcomes in male and female patients with COVID-19^a^.

Variables	Female (n = 1305)	Male (n = 1196)	*P* value
Age, median (IQR), year	56(40-67)	59(44-69)	0.0002^b^
Days from symptom onset to admission, median (IQR), (Missing=20)	9(5-15)	9(5-15)	0.2708^b^
Length of stay, median (IQR)	16(11-25)	16(11-26)	0.5095^b^
**Comorbidities**			
Any	588(45.1)	646(54.0)	<0.0001
Hypertension	418(32.0)	457(38.2)	0.0012
Diabetes	204(15.6)	213(17.8)	0.1445
Coronary heart disease	90(6.9)	145(12.1)	<0.0001
Cerebrovascular disease	62(4.8)	107(8.9)	<0.0001
COPD	64(4.9)	109(9.1)	<0.0001
Malignancy	90(6.9)	67(5.6)	0.1824
Chronic liver disease	78(6.0)	89(7.4)	0.1428
Chronic kidney disease	50(3.8)	87(7.3)	0.0002
**Signs and symptoms**			
Fever	806(61.8)	795(66.5)	0.0142
Dry cough	661(50.7)	562(47.0)	0.0673
Shortness of breath	284(21.8)	255(21.3)	0.7886
Fatigue	239(18.3)	224(18.7)	0.7896
Chest stuffiness	233(17.9)	217(18.1)	0.8507
Expectoration	184(14.1)	171(14.3)	0.8873
Anorexia	144(11.0)	120(10.0)	0.4157
Myalgia	71(5.4)	63(5.3)	0.8478
Diarrhea	75(5.7)	45(3.8)	0.0204
Vomiting	26(2.0)	26(2.2)	0.7506
**Chest computed tomography findings** (Missing=242)			0.0810
Ground-glass opacity	165(13.9)	145(13.5)	
Local patchy shadowing	162(13.6)	114(10.6)	
Bilateral patchy shadowing	861(72.5)	812(75.8)	
**Complications**			
Shock	13(1.0)	25(2.1)	0.0255
Acute respiratory distress syndrome	99(7.6)	135(11.3)	0.0015
Acute cardiac injury (Missing=913)	127(15.4)	165(21.6)	0.0015
Acute kidney injury (Missing=31)	369(28.7)	271(22.9)	0.0009
Secondary infection (Missing=568)	303(30.2)	370(39.8)	<0.0001
Urinary tract infection	116(22.9)	75(17.4)	0.0365
**Disease severity**			
Severe	562(43.1)	656(54.9)	<0.0001
**Clinical outcome**			<0.0001
Discharged	1238(94.9)	1068(89.3)	
Died	67(5.1)	128(10.7)	

COPD, Chronic obstructive pulmonary disease.

a Unless otherwise indicated, values shown are n(%).

b These P values are associated with Wilcoxon-Mann-Whitney-Test; all other P values are associated with χ^2^ tests.

**Supplementary Table 1** further showed the subgroup analysis of clinical characteristics. The prevalence of coronary heart disease, cerebrovascular disease, COPD, and chronic kidney disease among postmenopausal female patients were significantly lower than that of their age-matched male patients. There was a significant difference in the prevalence of hypertension between premenopausal female and their age-matched male patients. Females in postmenopausal group reported significantly higher prevalence of hypertension, diabetes, coronary heart disease, cerebrovascular disease, COPD, malignancy, chronic liver disease, and chronic kidney diseases compared to the premenopausal group. Propensity score matching (PSM) was performed to avoid age interference in the association between estrogen and disease outcomes. After PSM, 74 females in premenopausal group were matched at a 1:1 ratio to 74 females in postmenopausal group. There was no significant difference between the two groups in terms of clinical characteristics (**Supplementary Table 2**).

Of 2259 patients who underwent chest CT on admission, 165(13.9%) of females and 145(13.5%) of males showed ground-glass opacity, 162(13.6%) of females and 114(10.6%) of males showed unilateral pneumonia, and 861(72.5%) of females and 812(75.8%) of males showed bilateral pneumonia. There was no difference between the female and male groups (*P*=0.0810) (**Table 1**).

### Laboratory Findings of Female and Male Patients

In terms of laboratory findings on admission, male patients had more lymphopenia (455[38.2%] *vs.* 410[31.8%], *P*=0.0008) and thrombocytopenia (145[12.2%] *vs.* 87[6.7%], *P*<0.0001) as compared with female patients. Male patients also showed a higher inflammatory response (C-reactive protein: 710[61.1%] *vs.* 624[49.5%], *P*<0.0001; procalcitonin: 62[8.3%] *vs.* 28[3.3%], *P*<0.0001; interleukin-6: 123[45.2%] *vs.* 99[30.9%], *P*=0.0003) and were more prone to have hypoproteinemia (albumin: 568[48.0%] *vs.* 544[42.4%], *P*=0.0054) and extrapulmonary organ damage, such as cardiac injury (creatine kinase: 193[18.4%] *vs.* 103[9.0%], *P*<0.0001; high-sensitivity troponin I: 119[15.6%] *vs.* 99[12.0%], *P*=0.0394; myohemoglobin: 77[19.3%] *vs.* 48[11.7%], *P*=0.0028) and liver injury (alanine aminotransferase: 258[21.8%] *vs.* 152[11.9%], *P*<0.0001; aspartate aminotransferase: 173[17.7%] *vs.* 130[12.3%], *P*=0.0006; total bilirubin: 95[8.1%] *vs.* 42[3.3%], *P*<0.0001) (**Table 2**). The absolute values of the median and interquartile range (IQR) of laboratory findings were also shown in **Supplementary Table 3**. Normal ranges of each laboratory findings were listed in **Supplementary Table 4**.

**Table 2:** Laboratory findings in female and male patients with COVID-19 on admission.

**Variables**	**Female (n = 1305)**	**Male (n = 1196)**	***P* value****^a^**
**Hematologic**			
Blood leukocyte count >10×10^9^/L	94(7.3)	105(8.8)	0.1630
Lymphocyte count <1.1×10^9^/L	410(31.8)	455(38.2)	0.0008
Neutrophil count >6.3×10^9^/L	151(11.7)	154(12.9)	0.3533
Platelet count <125×10^9^/L	87(6.7)	145(12.2)	<0.0001
**Biochemical**			
Hemoglobin <110 g/L	292(22.6)	269(22.6)	0.9675
Alanine aminotransferase >50U/L	152(11.9)	258(21.8)	<0.0001
Aspartate aminotransferase >40U/L	130(12.3)	173(17.7)	0.0006
Lactate dehydrogenase >225U/L	247(24.4)	242(25.9)	0.4458
Total bilirubin >21μmol/L	42(3.3)	95(8.1)	<0.0001
Albumin <35g/L	544(42.4)	568(48.0)	0.0054
Blood urea >8.2mmol/L	95(7.4)	139(11.7)	0.0002
Creatinine >133μmol/L	208(16.2)	186(15.7)	0.7394
Creatine kinase >190U/L	103(9.0)	193(18.4)	<0.0001
Creatine kinase-MB >6.73ng/ml	10(4.3)	14(6.3)	0.3424
High-sensitivity troponin I >0.014ng/ml (99^th^ percentile)	99(12.0)	119(15.6)	0.0394
Myohemoglobin >75ng/ml	48(11.7)	77(19.3)	0.0028
Brain natriuretic peptide >100pg/ml	157(30.2)	169(35.7)	0.0670
**Coagulation test**			
Prothrombin time >15s	146(12.0)	228(20.0)	<0.0001
Activated partial thromboplastin time >40s	51(4.2)	58(5.1)	0.2939
D-dimer >1ug/ml	476(38.7)	457(40.5)	0.3773
**Infection-related indices**			
C-reactive protein >5 mg/L	624(49.5)	710(61.1)	<0.0001
Procalcitonin ≥0.5 ng/ml	28(3.3)	62(8.3)	<0.0001
Interleukin-6 >7pg/ml	99(30.9)	123(45.2)	0.0003

a All p-values are associated with χ2 tests.

Subgroup analysis of laboratory findings indicated that postmenopausal females had a lower incidence of lymphopenia, thrombocytopenia, and hypoproteinemia than age-matched males. The serum markers indicated that postmenopausal females are less likely to have elevated inflammatory response, disturbed coagulation function, and extrapulmonary organ damage, including cardiac injury and liver injury, than age-matched males. Premenopausal female showed weaker inflammatory response and liver injury than age-matched male patients (**Supplementary Table 5**).

### Complications and Outcomes of Female and Male Patients

Compared to female patients, male patients had a higher proportion of severe cases (656[54.9%] *vs.* 562[43.1%], *P <*0.0001) and deaths (128[10.7%] *vs.* 67[5.1%], *P*<0.0001). During hospitalization, male patients were more likely to have shock (25[2.1%] *vs.* 13[1.0%], *P*=0.0255), ARDS (135[11.3] *vs.* 99[7.6%], *P*=0.0015), acute cardiac injury (165[21.6%] *vs.* 127[15.4%], *P*=0.0015), secondary bacterial infections (370[39.8%] *vs.* 303 [30.2%], *P*<0.0001). Female patients were more prone to have acute kidney injury (369[28.7%] *vs.* 271[22.9%], *P*=0.0009) and urinary tract infection (116[22.9%] *vs.* 75[17.4%], *P*=0.0365) (**Table 1**)

Multivariable logistic regression analysis indicated that males were more likely to progress into severe type (OR=1.46; 95%CI: 1.24-1.73) and prone to ARDS (OR=1.37; 95%CI: 1.03-1.83), secondary bacterial infections (OR=1.39; 95%CI: 1.14-1.69) and death (OR=1.90; 95%CI: 1.36-2.66), but had a lower probability of acute kidney injury (OR=0.57; 95%CI: 0.47-0.70) and urinary tract infection (OR=0.60; 95%CI: 0.43-0.85) (**Table 3**). After further subgrouping the patients by disease severity, the incidence of secondary bacterial infections was also significantly higher in male patients with severe type of COVID-19 than in females (**Supplementary Table 6**)

**Table 3:** Multivariable logistic regression analysis of associations of groups with outcomes^a^.

Variables	Male *vs.* Female^b^	Postmenopausal females *vs.* Premenopausal females^b^	Males ≥50 years *vs.* Postmenopausal females^b^	Males <50 years *vs.* Premenopausal females^b^
**Complications**				
Shock	1.68(0.81-3.47)	0.66(0.06-6.98)	1.75(0.82-3.73)	0.91(0.06-15.00)
Acute respiratory distress syndrome	1.37(1.03-1.83)^*^	1.82(0.83-4.01)	1.36(1.00-1.83)^*^	1.42(0.61-3.34)
Acute cardiac injury (Missing=913)	1.25(0.94-1.68)	0.87(0.43-1.74)	1.36(0.99-1.86)	0.74(0.34-1.64)
Acute kidney injury (Missing=31)	0.57(0.47-0.70)^***^	0.91(0.61-1.36)	0.59(0.46-0.75)^***^	0.54(0.37-0.77)^**^
Secondary infection (Missing=568)	1.39(1.14-1.69)^**^	0.46(0.30-0.70)^**^	1.67(1.31-2.12)^***^	0.97(0.69-1.37)
Urinary tract infection	0.60(0.43-0.85)^**^	2.33(1.14-4.75)^*^	0.43(0.29-0.66)^***^	1.35(0.72-2.51)
**Disease severity**				
Severe	1.46(1.24-1.73)^***^	1.09(0.77-1.56)	1.37(1.11-1.68)^**^	1.65(1.23-2.22)^**^
**Clinical outcome**				
Died	1.90(1.36-2.66)^**^	2.05(0.59-7.15)	1.89(1.34-2.67)^**^	1.46(0.33-6.46)

***P < 0.0001, **P < 0.01, *P < 0.05.

a Adjusted for age and comorbidities including hypertension, diabetes, coronary heart disease, cerebrovascular disease, chronic obstructive pulmonary disease, malignancy, chronic liver disease, and chronic kidney disease. Hospital was modeled as a random effect in the multivariable logistic regression.

b The reference.

In the subgroup analysis, male patients age-matched with postmenopausal females were more likely to progress into severe type (OR=1.37; 95%CI: 1.11-1.68), and prone to ARDS (OR=1.36; 95%CI: 1.00-1.83), secondary bacterial infection (OR=1.67; 95%CI: 1.31-2.12), and death (OR=1.89; 95%CI: 1.34-2.67) than postmenopausal females. But they had lower risk of acute renal injury (OR=0.59; 95%CI: 0.46-0.75) and urinary tract infection (OR=0.43; 95%CI: 0.29-0.66). Male patients age-matched with premenopausal females were more likely to progress into severe type (OR=1.65; 95%CI: 1.23-2.22), but were less likely to prone to renal injury (OR=0.54; 95%CI: 0.37-0.77) than premenopausal females. The postmenopausal females were more likely to have urinary tract infection (OR=2.33; 95%CI: 1.14-4.75), but had a lower probability of secondary bacterial infections (OR=0.46; 95%CI: 0.30-0.70) compared with premenopausal female patients (**Table 3**). However, these differences were not observed after PSM between premenopausal and postmenopausal females by age (**Supplementary Table 7**).

## Discussion

In the present study, we reported detailed sex-disaggregated data on COVID-19 and explored the effect of estrogen in the disease outcomes. We found differences in clinical characteristics and outcomes do exist between male and female patients. These sex-dependent differences were more pronounced in males who were age-matched to postmenopausal. Sex is a crucial variable in the prognosis of COVID-19.

Significant sex difference in severe disease and death of COVID-19 were observed in this study. Male patients were more likely to be severe cases and had a significantly higher proportion of death than females, which is consistent with the findings of SARS-CoV (Channappanavar et al., 2017) and the MERS-CoV (Matsuyama et al., 2016). This may be related to the different respond of females and males to many virus infections (Kadel and Kovats, 2018). Females usually have stronger innate and adaptive immune responses and are relatively resistant to viral infections. Instead, males generate less robust immune responses and are more susceptible to infection (Bouman et al., 2005; Rettew et al., 2008; Klein and Flanagan, 2016). The X chromosome, sex hormones and differential expression of disease susceptibility genes between sexes may be involved in these sex-specific differences following virus infections (Schurz et al., 2019). The X chromosome contains a large number of immune-related genes. Therefore, females can clear pathogens faster and induce vaccine effectiveness greater than males (Schurz et al., 2019). Moreover, as a functional receptor for SARS-CoV-2, ACE2 plays an important role in the pathogenesis. Plasma concentrations of ACE2, have been found higher in male than in female with heart failure (Sama et al., 2020). This may be the addition reason for the differences in virus loads, tissue damage and outcomes between the sexes. In a mouse model of SARS-CoV infection, oophorectomy or estrogen receptor antagonist treatment increased mortality in female mice (Channappanavar et al., 2017). However, there was no significant difference in disease severity and mortality between postmenopausal and premenopausal females after eliminating the confounding of age in this study. A retrospective study in patients with COVID-19 in China also reported that estrogen might not be directly related to the lower mortality in females (Sha et al., 2021). Due to the small sample size after PSM, large sample investigations are still needed to verify this conclusion.

Male patients were also more prone to severe complications. Besides more severe inflammatory immune response aforementioned, more pre-existing diseases (i.e., hypertension, coronary heart disease, cerebrovascular disease, COPD, and chronic kidney disease) in male patients may be an additional reason. Current evidence from Wuhan, China has shown that the COVID-19 mortality rate is comorbidity-dependent, and the crude case mortality rate increased to 10.5%, 7.3% and 6.3% in patients with cardiovascular disease, diabetes mellitus or hypertension, respectively (Epidemiology Working Group for NCIP Epidemic Response and Chinese Center for Disease Control and Prevention, 2020). The presence of these comorbidities may put male patients in weaker immune functions and further aggravated hyperinflammatory state after SARS-CoV-2 infection, leading to more severe multiple organ damage. Pre-existing diseases usually cause organs damage, which are also more likely to deteriorate when SARS-CoV-2 infection occurs. Furthermore, effects of comorbidities on ACE2 expression and activities should be considered (Sama et al., 2020). We also found secondary infection was more common in male patients than in females, and the sex difference remained significant in severe cases. The results of a recent study also showed that males in severe illness were more likely to develop secondary infection (Su et al., 2020). It is suggested that the incidence of secondary infection in severe cases should be closely monitored by clinicians and reported as a complication, especially in male patients.

Previous studies showed that AKI was more common in male patients compared to females (Su et al., 2020; Vahidy et al., 2021). However, our study found that the incidence of AKI was higher in females than males. The reason may be due to the fact that on the one hand, gastrointestinal symptoms, especially diarrhea, are more common in females, and differences in care for dehydration will affect the frequency of AKI. Therefore, the higher incidence of AKI in females may be related to pre-renal acute kidney injury. On the other hand, urinary tract infections were more common in females, which may also contribute to the higher incidence of AKI in females than males in this study. Furthermore, since the incidence of AKI may vary depending on the diagnostic criteria (Luo et al., 2014), this may also be one reason why the present findings differ from other studies. Clinical trials with large samples are still needed to explore the causes of the different incidence of AKI in male and female patients with COVID-19.

The subgroup results showed that males that age-matched with postmenopausal females were more common in severe illness and prone to death. Data from Global Health 5050 also indicated that the overall case fatality ratio in males is indeed higher than females (Global Health 50/50). Thus, combined with recent studies (Chen et al., 2020; Dangis et al., 2020; Guan et al., 2020), we believed that sex is a crucial variable in the prognosis. Male sex, especially those aged over 50 years old, is related with the severe disease and death from SARS-Cov-2 infection.

Our study has several limitations. First, some laboratory tests (i.e., high-sensitivity troponin I, N-terminal pro-brain natriuretic peptide, creatinine, and cytokine level measurements) were not done in all the patients, and missing data might lead to bias of results. Second, due to all patients included in this study being adults, it was hard to assess whether sex-specific differences in clinical characteristics and disease outcomes also existed among younger age groups. Future studies should pay more attention to COVID-19 patients younger than 18 years, such as adolescents, to fill these gaps.

## Conclusions

In conclusion, collecting sex-disaggregated data is essential to understanding the feature of COVID-19, the risk factors of poor prognosis, and developing the strategy of treatment. Here, we reported detailed sex-disaggregated data on SARS-CoV-2 infection and confirmed that sex is a crucial variable in the clinical characteristics and outcomes. A comprehensive management plan with a sex perspective is necessary. Males, especially those age-matched with postmenopausal females, require additional prevention, surveillance, or earlier intensive intervention. Estrogen may not be the primary reason to the sex-specific differences in disease outcomes.

## Data Availability Statement

The original contributions presented in the study are included in the article/**Supplementary Material**. Further inquiries can be directed to the corresponding author.

## Ethics Statement

This study was approved by the Ethics Committee of Tongji Medical College, Huazhong University of Science and Technology. Written informed consent for participation was not required for this study in accordance with the national legislation and the institutional requirements.

## Author Contributions

MW, NJ, CL, and GZ were responsible for the conception, design, and writing of the manuscript. CL, HY, LL, XT, YG, and XY were responsible for the acquisition of data and literature research. MW, NJ, JW, ZC, QZ, NX were responsible for the analysis and interpretation of data. All authors contributed to the article and approved the submitted version. The corresponding author attests that all listed authors meet authorship criteria and that no others meeting the criteria have been omitted.

## Funding

This study was funded by the Fundamental Research Funds for the Central Universities, Huazhong University of Science and Technology (No. 2020kfyXGYJ073).

## Conflict of Interest

The authors declare that the research was conducted in the absence of any commercial or financial relationships that could be construed as a potential conflict of interest.
