# Supplementary material for: Sex-Disaggregated Data on Clinical Characteristics and Outcomes of Hospitalized Patients With COVID-19: A Retrospective Study
Source: Front Cell Infect Microbiol. 2021 May 26;11:680422. doi: 10.3389/fcimb.2021.680422 (PMC8187910; doi:10.3389/fcimb.2021.680422)
Supplement: Supplementary file 1 [file Table_1.docx]

**Supplementary Table 1.** Clinical characteristics on admission in different subgroups of male and female patients with COVID-19.

| Variables | Female | |  | Male | |  | |
| --- | --- | --- | --- | --- | --- | --- | --- |
|  | Premenopausal (n=494) | Postmenopausal (n=811) |  | <50 years (n=393) | ≥50 years (n=803) | |  |
| Age, median (IQR), year | 36(29-42) | 65(57-72) ^a^ |  | 38(33-44) ^b^ | 65(58-73) | |  |
| **Comorbidities** |  |  |  |  |  | |  |
| Any | 109(22.1) | 541(66.7) ^a^ |  | 111(28.2) ^b^ | 593(73.9) ^c^ | |  |
| Hypertension | 37(7.5) | 381(47.0) ^a^ |  | 47(12.0) ^b^ | 410(51.1) | |  |
| Diabetes | 24(4.9) | 180(22.2) ^a^ |  | 18(4.6) | 195(24.3) | |  |
| Coronary heart disease | 2(0.4) | 88(10.9) ^a^ |  | 5(1.3) | 140(17.4) ^c^ | |  |
| Cerebrovascular disease | 1(0.2) | 61(7.5) ^a^ |  | 2(0.5) | 105(13.1) ^c^ | |  |
| Chronic obstructive pulmonary disease | 8(1.6) | 56(6.9) ^a^ |  | 13(3.3) | 96(12.0) ^c^ | |  |
| Malignancy | 16(3.2) | 74(9.1) ^a^ |  | 10(2.5) | 57(7.1) | |  |
| Chronic liver disease | 38(7.7) | 40(4.9) ^a^ |  | 37(9.4) | 52(6.5) | |  |
| Chronic kidney disease | 5(1.0) | 45(5.6) ^a^ |  | 8(2.0) | 79(9.8) ^c^ | |  |

IQR, interquartile range.

^a^ There are statistically significant differences in corresponding indicators differences between premenopausal and postmenopausal females with COVID-19.

^b^ There are statistically significant differences in corresponding indicators differences between premenopausal females and age-matched males with COVID-19.

^c^ There are statistically significant differences in corresponding indicators differences between postmenopausal females and age-matched males with COVID-19.**Supplementary Table 2.** The clinical characteristics of female after age-matched between premenopausal and postmenopausal group.

| Variables | Females | | |
| --- | --- | --- | --- |
|  | Premenopausal (n=74) | Postmenopausal (n=74) | *P* value |
| Age, median (IQR), years | 49(47-51) | 50(47-52) | 0.0999 |
| **Comorbidities** |  |  |  |
| Any | 26(35.1) | 29(39.2) | 0.6098 |
| Hypertension | 12(16.2) | 18(24.3) | 0.2199 |
| Diabetes | 10(13.5) | 6(8.1) | 0.2897 |
| Coronary heart disease | 1(1.4) | 1(1.4) | 1.0000 |
| Cerebrovascular disease | 0(0.0) | 2(2.7) | 0.4765 |
| Chronic obstructive pulmonary disease | 3(4.1) | 0(0.0) | 0.2434 |
| Malignancy | 4(5.4) | 4(5.4) | 1.0000 |
| Chronic liver disease | 3(4.1) | 5(6.8) | 0.7162 |
| Chronic kidney disease | 2(2.7) | 2(2.7) | 1.0000 |
| **Signs and symptoms** |  |  |  |
| Fever | 56(75.7) | 51(68.9) | 0.3584 |
| Dry cough | 37(50.0) | 48(64.9) | 0.0674 |
| Shortness of breath | 15(20.3) | 20(27.0) | 0.3334 |
| Fatigue | 15(20.3) | 16(21.6) | 0.8399 |
| Chest stuffiness | 13(17.6) | 21(28.4) | 0.1180 |
| Expectoration | 10(13.5) | 19(25.7) | 0.0623 |
| Anorexia | 6(8.1) | 14(18.9) | 0.0544 |
| Myalgia | 3(4.1) | 5(6.8) | 0.7162 |
| Diarrhea | 4(5.4) | 7(9.5) | 0.3471 |
| Vomiting | 2(2.7) | 3(4.1) | 1.0000 |

IQR, interquartile range.

**Supplementary Table 3.** Absolute values of laboratory findings on admission in female and male patients with COVID-19.

| Variables | Female(n=1305) | Male(n=1196) | *P* value |
| --- | --- | --- | --- |
| **Hematologic** |  |  |  |
| Blood leukocyte count, median (IQR), 10^9^/L | 5.2(4.1-6.8) | 5.7(4.4-7.1) | <0.0001 |
| Lymphocyte count, median (IQR), 10^9^/L | 1.3(0.9-1.7) | 1.1(0.8-1.6) | <0.0001 |
| Neutrophil count, median (IQR), 10^9^/L | 3.4(2.3-4.7) | 3.6(2.8-5.2) | <0.0001 |
| Platelet count, median (IQR), 10^9^/L | 201.0(161.0-255.0) | 180.0(138.5-236.0) | <0.0001 |
| **Biochemical** |  |  |  |
| Haemoglobin, median (IQR), g/L | 124.0(114.0-132.0) | 138.0(126.0-149.0) | <0.0001 |
| Alanine aminotransferase, median (IQR), U/L | 16.7(11.4-25.8) | 26.1(16.2-40.9) | <0.0001 |
| Aspartate aminotransferase, median (IQR), U/L | 19.7(15.6-27.1) | 24.2(18.0-35.2) | <0.0001 |
| Lactate dehydrogenase, median (IQR), U/L | 174.0(141.7-231.0) | 191.0(152.0-258.0) | <0.0001 |
| Total bilirubin, median (IQR), μmol/L | 8.3(6.0-11.5) | 10.3(7.2-14.4) | <0.0001 |
| Albumin, median (IQR), g/L | 39.1(35.2-42.4) | 38.1(34.3-41.8) | 0.0005 |
| Blood urea, median (IQR), mmol/L | 3.8(3.0-5.1) | 4.8(3.8-6.6) | <0.0001 |
| Creatinine, median (IQR), μmol/L | 57.9(49.1-70.9) | 78.3(66.5-93.0) | <0.0001 |
| Creatine kinase, median (IQR), U/L | 53.0(36.0-85.0) | 82.0(51.4-141.0) | <0.0001 |
| Creatine kinase-MB, median (IQR), ng/ml | 0.9(0.5-1.6) | 1.1(0.7-2.2) | 0.0101 |
| High-sensitivity troponin I, median (IQR), ng/ml | 0.009(0.003-0.026) | 0.013(0.005-0.030) | <0.0001 |
| Myohemoglobin, median (IQR), ng/ml | 16.1(8.0-32.7) | 28.2(15.1-71.3) | <0.0001 |
| Brain natriuretic peptide, median (IQR), pg/ml | 55.0(19.5-140.4) | 64.0(19.0-174.6) | 0.2267 |
| **Coagulation test** |  |  |  |
| Prothrombin time, median (IQR), s | 11.6(11.0-12.3) | 11.9(11.3-12.9) | <0.0001 |
| Activated partial thromboplastin time, median (IQR), s | 27.0(24.0-30.6) | 28.1(24.9-32.3) | <0.0001 |
| D-dimer, median (IQR), median (IQR), ug/ml | 0.6(0.3-1.4) | 0.6(0.3-1.5) | 0.1709 |
| **Infection-related indices** |  |  |  |
| C-reactive protein, median (IQR), mg/L | 6.1(1.0-31.3) | 16.1(2.5-53.2) | <0.0001 |
| Procalcitonin, median (IQR), ng/ml | 0.05(0.04-0.07) | 0.06(0.05-0.12) | <0.0001 |
| Interleukin-6, median (IQR), pg/ml | 3.8(2.0-9.8) | 5.7(2.4-23.5) | 0.0002 |

IQR, interquartile range.

**Supplementary Table 4.** The normal range of laboratory indicators in this study.

| Variables | Normal range | Units |
| --- | --- | --- |
| **Hematologic** |  |  |
| Blood leukocyte count | 4-10 | 10^9^/L |
| Lymphocyte count | 1.1-3.2 | 10^9^/L |
| Neutrophil count | 1.8-6.3 | 10^9^/L |
| Platelet count | 125-350 | 10^9^/L |
| **Biochemical** |  |  |
| Haemoglobin | 110-172 | g/L |
| Alanine aminotransferase | 9-50 | U/L |
| Aspartate aminotransferase | 0-40 | U/L |
| Lactate dehydrogenase | 135-225 | U/L |
| Total bilirubin | 2-21 | μmol/L |
| Albumin | 35-55 | g/L |
| Blood urea | 1.7-8.2 | mmol/L |
| Creatinine | 20-133 | μmol/L |
| Creatine kinase | 0-190 | U/L |
| Creatine kinase-MB | 0-6.73 | ng/ml |
| High-sensitivity troponin I | 0-0.014 | ng/ml |
| Myohemoglobin | 25-75 | ng/ml |
| Brain natriuretic peptide | 0-100 | pg/ml |
| **Coagulation test** |  |  |
| Prothrombin time | 9-15 | s |
| Activated partial thromboplastin time | 20-40 | s |
| D-dimer | 0-1 | ug/ml |
| **Infection-related indices** |  |  |
| C-reactive protein | 0-5 | mg/L |
| Procalcitonin | 0-0.05 | ng/ml |
| Interleukin-6 | 0-7 | pg/ml |

**Supplementary Table 5.** Laboratory findings on admission in different subgroups of male and female patients with COVID-19.

| Variables | Female | |  | Male | |  |
| --- | --- | --- | --- | --- | --- | --- |
|  | Premenopausal (n=494) | Postmenopausal (n=811) |  | ＜50 years (n=393) | ≥50 years (n=803) |  |
| **Hematologic** |  |  |  |  |  |  |
| Blood leukocyte count >10×10^9^/L | 32(6.6) | 62(7.7) |  | 28(7.1) | 77(9.6) |  |
| Lymphocyte count <1.1×10^9^/L | 112(23.1) | 298(37.1) ^a^ |  | 100(25.6) | 355(44.4) ^c^ |  |
| Neutrophil count >6.3×10^9^/L | 41(8.4) | 110(13.7) ^a^ |  | 41(10.5) | 113(14.1) |  |
| Platelet count <125×10^9^/L | 28(5.8) | 59(7.3) |  | 37(9.4) ^b^ | 108(13.5) ^c^ |  |
| **Biochemical** |  |  |  |  |  |  |
| Haemoglobin <110g/L | 84(17.3) | 208(25.9) ^a^ |  | 31(7.9) ^b^ | 238(29.8) |  |
| Alanine aminotransferase >50U/L | 51(10.6) | 101(12.6) |  | 112(28.9) ^b^ | 146(18.3) ^c^ |  |
| Aspartate aminotransferase >40U/L | 31(7.7) | 99(15.1) ^a^ |  | 51(15.6) ^b^ | 122(18.7) |  |
| Lactate dehydrogenase >225U/L | 45(11.9) | 202(31.8) ^a^ |  | 57(19.2) ^b^ | 185(29.0) |  |
| Total bilirubin >21μmol/L | 15(3.1) | 27(3.4) |  | 26(6.7) ^b^ | 69(8.7) ^c^ |  |
| Albumin <35g/L | 138(28.6) | 406(50.8) ^a^ |  | 91(23.5) | 477(59.9) ^c^ |  |
| Blood urea >8.2mmol/L | 14(2.9) | 81(10.1) ^a^ |  | 18(4.7) | 121(15.1) ^c^ |  |
| Creatinine >133μmol/L | 55(11.5) | 153(19.0) ^a^ |  | 43(11.1) | 143(17.9) |  |
| Creatine kinase >190U/L | 23(5.4) | 80(11.1) ^a^ |  | 61(18.2) ^b^ | 132(18.4) ^c^ |  |
| Creatine kinase-MB >6.73ng/ml | 1(1.6) | 9(5.3) |  | 2(3.8) | 12(7.1) |  |
| High-sensitivity troponin I >0.014ng/ml (99^th^ percentile) | 14(5.3) | 86(15.3) ^a^ |  | 10(4.7) | 110(20.0) ^c^ |  |
| Myohemoglobin >75ng/ml | 1(0.9) | 47(15.7) ^a^ |  | 12(12.0) ^b^ | 65(21.7) |  |
| Brain natriuretic peptide >100pg/ml | 18(11.3) | 139(38.5) ^a^ |  | 11(9.2) | 158(44.5) |  |
| **Coagulation test** |  |  |  |  |  |  |
| Prothrombin time >15s | 34(7.7) | 112(14.4) ^a^ |  | 35(9.7) | 193(24.8) ^c^ |  |
| Activated partial thromboplastin time >40s | 17(3.9) | 34(4.4) |  | 12(3.3) | 46(5.9) |  |
| D-dimer >1ug/ml | 103(22.6) | 373(48.1) ^a^ |  | 79(22.3) | 378(48.8) |  |
| **Infection-related indices** |  |  |  |  |  |  |
| C-reactive protein >5 mg/L | 162(34.2) | 462(58.6) ^a^ |  | 192(49.9) ^b^ | 518(66.6) ^c^ |  |
| Procalcitonin ≥0.5 ng/ml | 2(0.6) | 26(5.1) ^a^ |  | 10(4.0) ^b^ | 52(10.4) ^c^ |  |
| Interleukin-6 >7pg/ml | 21(18.3) | 78(38.1) ^a^ |  | 22(27.9) | 101(52.3) ^c^ |  |

^a^ There are statistically significant differences in corresponding indicators differences between premenopausal and postmenopausal females with COVID-19.
^b^ There are statistically significant differences in corresponding indicators differences between premenopausal females and age-matched males with COVID-19.
^c^ There are statistically significant differences in corresponding indicators differences between postmenopausal females and age-matched males with COVID-19.

**Supplementary Table 6.** Multivariable logistic regression analysis of association of groups with secondary infection in severe and non-severe patients^a^.

| Variables | Male vs Female^b^ | Postmenopausal females vs. Premenopausal females^b^ | Males ≥50 years vs.  Postmenopausal females^b^ | Males <50 years vs. Premenopausal females^b^ |
| --- | --- | --- | --- | --- |
| **Severity** |  |  |  |  |
| Severe | 1.44(1.12-1.86)^**^ | 0.62(0.36-1.06) | 1.65(1.24-2.22)^**^ | 0.97(0.57-1.66) |
| Non-severe | 1.06(0.76-1.47) | 0.32(0.19-0.55)^***^ | 1.30(0.81-2.10) | 0.89(0.56-1.42) |

^***^*P*<0.0001, ^**^*P*<0.01, ^*^*P*<0.05

^a^Adjusted for age and comorbidities including hypertension, diabetes, coronary heart disease, cerebrovascular disease, chronic obstructive pulmonary disease, malignancy, chronic liver disease, and chronic kidney disease. Hospital was modeled as a random effect in the multivariable logistic regression.

^b^The reference.

**Supplementary Table 7.** Multivariable logistic regression analysis of outcomes in postmenopausal and premenopausal females^a^.

| Variables | Postmenopausal females vs. Premenopausal females^b^ |
| --- | --- |
| **Complications** |  |
| Shock | - |
| Acute respiratory distress syndrome | 0.43(0.10-1.87) |
| Acute cardiac injury | 1.94(0.45-8.41) |
| Acute kidney injury | 1.03(0.45-2.36) |
| Secondary infection | 0.46(0.18-1.16) |
| Urinary tract infection | 1.28(0.28-5.91) |
| **Disease severity** |  |
| Severe | 0.63(0.32-1.24) |
| **Clinical outcome** |  |
| Died | 1.83(0.16-21.5) |

^a^Adjusted for age and hypertension. Hospital was modeled as a random effect in the multivariable logistic regression.

^b^The reference.

-The shock between two groups only has one level.
